# Supplementary material for: Macrophages discriminate and sort pathogenic bacteria and apoptotic material during dual-target phagocytosis
Source: PLoS One. 2026 Oct 7;21(10):e0356679. doi: 10.1371/journal.pone.0356679 (PMC13645013; doi:10.1371/journal.pone.0356679)
Supplement: Supplementary file 1 [file pone.0356679.s001.pdf]

A

B

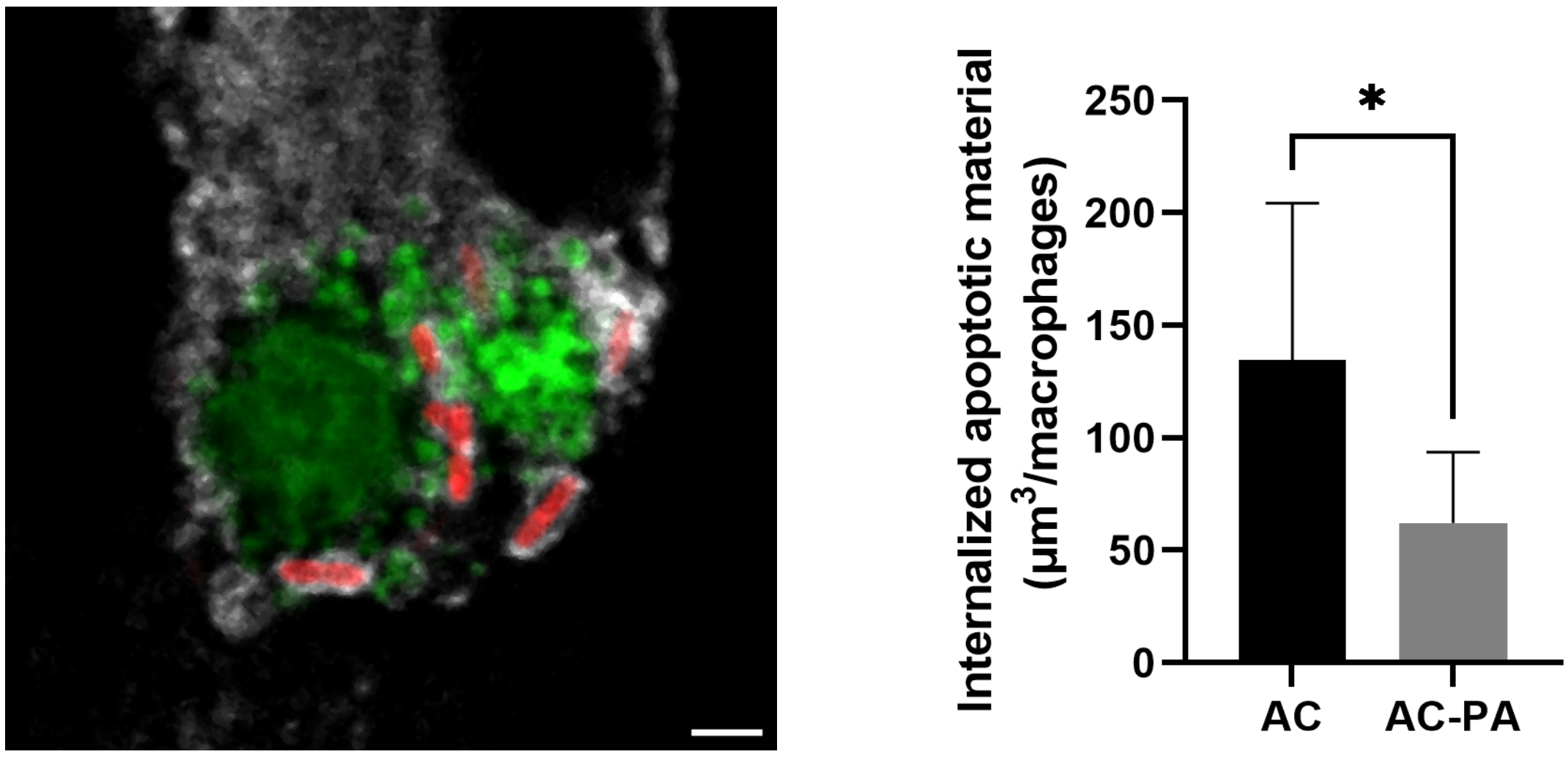

Internalized apoptotic material
(µm3/macrophages)
250
200
150
100
50
0
*
AC
AC-PA
